# Contribution of chronic diseases to the disability burden in a population 15 years and older, Belgium, 1997–2008

**DOI:** 10.1186/s12889-015-1574-z

**Published:** 2015-03-07

**Authors:** Renata TC Yokota, Nicolas Berger, Wilma J Nusselder, Jean-Marie Robine, Jean Tafforeau, Patrick Deboosere, Herman Van Oyen

**Affiliations:** Department of Public Health and Surveillance, Scientific Institute of Public Health, Rue Juliette Wytsmanstraat 14, 1050 Brussels, Belgium; Department of Social Research, Interface Demography, Vrije Universiteit Brussel, 1050 Brussels, Belgium; Department of Social & Environmental Health Research, London School of Hygiene & Tropical Medicine, London, UK; Department of Public Health, Erasmus MC, Rotterdam, The Netherlands; French Institute of Health and Medical Research (INSERM), Montpellier, France; École Pratique des Hautes Études, Paris, France; Department of Public Health, Ghent University, Ghent, Belgium

**Keywords:** Disability, Chronic disease, Belgium

## Abstract

**Background:**

Age-associated disability reduces quality of life in older populations and leads to wide-range implications for social and health policy. The identification of diseases that contribute to the disability burden is crucial to the development of prevention and intervention strategies to reduce disability. In this study, we assessed the contribution of chronic diseases to the prevalence of disability in Belgium.

**Methods:**

Data from 35,837 individuals aged 15 years or older who participated in the 1997, 2001, 2004, or 2008 Belgian Health Interview Surveys were used. Disability was defined as difficulties in doing at least one of six activities of daily living (transfer in and out of bed, transfer in and out of chair, dressing, washing hands and face, feeding, and going to the toilet) and/or mobility limitations (ability to walk without stopping less than 200 m). Multiple additive regression models were fitted separately for men and women to estimate the age-specific background disability rate (experienced by everyone, independent of the presence of specific diseases) and disease-specific disability rates (disability rate in subjects who reported selected chronic diseases).

**Results:**

Musculoskeletal, cardiovascular, and respiratory diseases were the main contributors to the disability burden in Belgium. Musculoskeletal diseases were the most prevalent diseases in men and women in all age groups. Neurological diseases and stroke were the most disabling diseases, i.e. caused the highest level of disability among the diseased individuals, in all age groups for men and women, respectively. Back pain was the main cause of disability in men aged 15 to 64 years, while heart attack was the major contributor to the disability prevalence in men aged 65 or older. Likewise, arthritis was the main cause of disability among women across all age groups. Depression was also an important contributor in young subjects (15–54 years). Cancer was not an important contributor to the disability prevalence in Belgium.

**Conclusions:**

To reduce the burden of disability in Belgium, interventions should target musculoskeletal, cardiovascular and respiratory diseases especially among elderly. Furthermore, attention should also be given to depression in young individuals.

**Electronic supplementary material:**

The online version of this article (doi:10.1186/s12889-015-1574-z) contains supplementary material, which is available to authorized users.

## Background

Global population aging accompanied by an increased longevity with disability has raised international concern, particularly due to its social and economic costs for health care systems [1]. The identification of diseases with greatest potential to influence the health of populations can assist public health policies in the definition of prevention and control strategies [2,3].

Although longitudinal studies are considered the gold standard in the assessment of disability causes, their use is often limited due to their high costs, which can result in restricted sample sizes and lack of representativeness of large populations [4]. Alternative methods have been proposed to assess the impact of specific diseases in the burden of disability using cross-sectional data [5-10]. The first methods were focused on the effect of elimination of specific diseases on mortality and disability [5-9]. However, the results depend upon the order a cause is removed, which can produce inconsistent results in the presence of comorbidity [11]. Also, these methods often rely on a multiplicative model (logistic regression) which does not yield additive contributions of diseases [11]. To overcome this limitation, the use of the average attributable fraction was proposed and recently applied on the estimation of disability by cause in France [12]. The main drawback of this approach is the use of the odds ratio (OR) as a measure of the relative risk, especially for diseases with high prevalence, which is the case of several chronic diseases [5]. When the OR is not used, the method relies on the availability of relative risks in the literature [5]. As an alternative, Nusselder and Looman [10,13] proposed a method based on the use of additive hazards models to attribute the total disability prevalence into cause-specific contributions of diseases and background in the presence of comorbidity [11]. In this approach, the background represents causes of disability not included in the analysis.

To date, only six studies using data from Belgium [13], The Netherlands [3,10,11], Germany [14] and China [15] estimated the disability prevalence by cause using the attribution method. In this study, the contribution of specific chronic diseases to the prevalence of disability in Belgium using the attribution method [10] is presented.

## Methods

### Study participants

Individuals aged 15 years or older who participated in the Belgian Health Interview Survey (HIS) of 1997, 2001, 2004 or 2008 were included in this study. The Belgian HIS is a household survey based on a representative, stratified (regions and provinces) and clustered (municipalities and households) sample of the Belgian population, including also elderly subjects admitted in institutions. Each survey included approximately 10,000 individuals. The response rate was 59% in 1997, 61% in 2001, 61% in 2004, and 55% in 2008. Sampling weights were used to take into account the complex sample design. Approximately 200 interviewers were necessary for the data collection in each HIS. All interviewers followed a training to ensure standardisation of data collection and field work procedures. The data collection included a face-to-face interview and a self-administered questionnaire. More details about the surveys methodology can be found in previous publications [16,17]. The Belgian HIS data is available upon request (https://his.wiv-isp.be/SitePages/Acces_microdata.aspx) and approval of the Belgian Commission of Privacy Protection. Informed consent was obtained from all survey participants. The data collection was carried out by Statistics Belgium and the surveys were exempted by law from requiring ethical approval.

The disability and disease/conditions questions were included in the face-to-face questionnaire in the four HIS. Since the disability questions were restricted to the population of 15 years or older, the analysis was also limited to this subsample. The data of the four HIS were pooled, resulting in 39,587 subjects. Due to lack of disease (n = 1,975) or disability information (n = 1,891), 3,750 (9%) individuals were excluded from the analysis, resulting in a sample of 35,837 subjects (7,928 in the 1997 HIS; 9,183 in the 2001 HIS; 9,996 in the 2004 HIS; and 8,730 in the 2008 HIS). More detailed information on the study participants is presented in Table 1. Women, elderly, and individuals with primary education were overrepresented in the excluded subjects (Additional file 1).

**Table 1:** **Characteristics of the study population, Belgium, 1997, 2001, 2004, and 2008**

**Characteristics**	**Men**								**Women**							
	**15-54**		**55-64**		**65-79**		**≥80**		**15-54**		**55-64**		**65-79**		**≥80**	
	**N**	%*	**N**	%*	**N**	%*	**N**	%*	**N**	%*	**N**	%*	**N**	%*	**N**	%*
Survey year																
1997	2735	24.4	450	20.3	541	21.3	108	10.3	2698	23.9	482	21.1	693	22.3	221	10.3
2001	3093	27.5	581	26.2	675	26.6	152	14.5	3031	26.9	598	26.2	826	26.6	227	10.6
2004	2848	25.4	585	26.4	811	32.0	385	36.6	2959	26.2	603	26.4	961	31.0	844	39.4
2008	2551	22.7	603	27.2	510	20.1	406	38.6	2590	23.0	597	26.2	624	20.1	849	39.7
Education level																
Tertiary	3895	34.7	772	34.8	693	27.3	266	25.3	4486	39.8	657	28.8	591	19.0	259	12.1
Secondary	4799	42.7	934	42.1	967	38.1	360	34.3	4134	36.7	1014	44.5	1213	39.1	736	34.4
Primary	732	6.5	435	19.6	756	29.8	357	34.0	770	6.8	511	22.4	1127	36.3	921	43.0
No diploma	72	0.6	37	1.7	54	2.1	27	2.6	106	0.9	41	1.8	85	2.7	92	4.3
No information	1729	15.4	41	1.8	67	2.6	41	3.9	1782	15.8	57	2.5	88	2.8	133	6.2
Disabled	415	3.7	249	11.2	631	24.9	527	50.1	534	4.7	326	14.3	1003	32.3	1453	67.9
Total	11227		2219		2537		1051		11278		2280		3104		2141	

*Not weighted.

### Definition of disability

Disability was defined in terms of self-reported difficulties in performing activities of daily living (ADL)–ability to transfer in-and-out of bed, transfer in-and-out of chair, dressing/undressing, washing hands and face, feeding, and using the toilet – and limitations on mobility. These questions were part of the recommended instrument to measure disability proposed by the World Health Organization [18]. The disability definition used in this study was based on the availability of the disability questions in the four HIS. An individual was considered disabled if he/she reported having difficulties in performing at least one of the ADLs on his/her own or only being able to perform it with personal assistance. An individual was also considered disabled if he/she reported an ability to walk without stopping that was less than 200 meters.

### Definition of chronic diseases

The HIS participants were asked about the presence of disease over the year preceding the interview. In total, 20 diseases or diseases groups were included, based on their availability in the four HIS waves: chronic respiratory diseases (asthma, chronic bronchitis, and chronic pulmonary diseases), diabetes, cancer, depression, chronic cystitis, heart attack, stroke, arthritis (rheumatoid arthritis and osteoarthritis), back pain, osteoporosis, stomach ulcer, bowel diseases, cirrhosis, gall-stones, cataract, glaucoma, migraine, thyroid problems, skin diseases, and neurological diseases (epilepsy and Parkinson’s disease). Other diseases and conditions, such as injuries and dementia, were not included in the analysis due to the lack of information in the four HIS.

### Statistical analysis

The attribution method was used to estimate the disability prevalence by cause, i.e. to attribute disability to disease and “background”, using cross-sectional data [10]. Analogous to the underlying cause of death, in which one disease is assigned as underlying cause of death according to the death certificate, we aim to attribute each disability case reported in the surveys to a single cause, taking into account that individuals can have more than one disease (comorbidity) and that disability can be present in individuals without any disease [4].

Even if an individual reports a disease in the survey, this is not necessarily the cause of the disability. This disability that is not associated with the diseases included in the analysis is labelled “background”. Disability in individuals who did not report any disease is entirely attributed to background, while disability in individuals who reported diseases is partitioned among the diseases and background [4].

The method assumes that (i) the distribution of disability by cause is entirely explained by diseases that are still present at the time of the survey and the background, (ii) the cause-specific disability rates for each disease were proportionally equal in the time preceding the survey, (iii) individuals from the same age groups are exposed to the same background rate, (iv) the causes of disability (diseases and background) act as independent competing causes, and (v) the start of the time at risk for disability is the same for all causes [4].

In the multiple decrement life table, under the assumption of independence between causes of death, an exponential transformation is applied to the cumulative force of mortality to obtain the cause-specific probability of death in the presence of competing risks [19-21]. Analogous to this method, we used hazards rates to obtain the probability of being disabled by cause. Under the additive assumption of the rates, the total disability rate can be obtained by adding up the cause-specific disability rates [21].

The attribution method is based on the multiple additive regression model [10] as shown in equation (1).

1 $$ \begin{array}{l}{Y}_i= Bernoulli\left({\pi}_i\right)\\ {}{\pi}_i=1-{e}^{-{\eta}_i}\\ {}{\eta}_i={\alpha}_a+{\displaystyle \sum_{d=1}^m}{\beta}_{cd}{X}_{di}\end{array} $$

Where:

- *Y*_*i*_ is the binary response (disability) variable for each individual *i*;
- *π*_*i*_ is the estimated probability that individual *i* is disabled;
- *e* is the base of the natural logarithm;
- *η*_*i*_ is the total disability rate (linear predictor) for each individual *i*;
- *α*_*a*_ is the background disability rate by age group *a*(1, …, *n*) (15–54, 55–59, 60–64, 65–69, 70–74, 75–79, ≥ 80 years);
- *β*_*cd*_ is the disease-specific disability rate (disabling impact). It is defined as the product *β*_*cd*_ = *γ*_*c*_ * *δ*_*d*_, where *γ*_*c*_ is the age pattern, which allows the disease prevalence to vary across age group *c*(1, …, *k*) (15–54, 55–64, 65–79, and ≥ 80 years), and δ_d_ is the disease effect, specific for each disease *d*(1, …, *m*);
- *X*_*di*_ is the indicator variable for each disease *d* and individual *i*.

Model (1) is a reduced rank regression with one rank [22], as the number of parameters estimated for the interaction between age and disease is reduced by assuming that the age pattern (*γ*_*c*_) varies across age groups but is similar across diseases and that the disease effect (*δ*_*d*_) differs across diseases, but not by age group. In other words, instead of estimating *m* * *k* (20 * 4 = 80) parameters, which represents the maximum number of two-way interaction terms between age and disease, we only estimate *m* + *k* (20 + 4 = 24).

The estimation of the disability prevalence by cause depends on the prevalence of the disease (*X*_*di*_) and the disabling impact of the disease across age group (*β*_*cd*_) [11]. The disabling impact represents the rate at which each chronic disease causes disability among the diseased individuals [23]. The attribution of disability to background and to disease *d* is defined in equation (2).

2 $$ \begin{array}{l}{B}_i=\frac{\alpha_a}{\eta_i}*{\pi}_i\\ {}{D}_{di}=\frac{\beta_{cd}\ {X}_{di}}{\eta_i}*{\pi}_i\end{array} $$

Where *B*_*i*_ is the probability of individual *i* to be disabled due to background and *D*_*di*_ is the probability of individual *i* to be disabled due to disease *d*.

The total number of disabled individuals by background and by each disease can be obtained as shown in equation (3).

3 $$ \begin{array}{l}{N}_b={\displaystyle \sum_{i=1}^N}{B}_i\\ {}{N}_d={\displaystyle \sum_{i=1}^N}{D}_{di}\end{array} $$

Where *N*_*b*_ is the total number of disabled individuals due to background and *N*_*d*_ is the total number of disabled individuals due to disease *d*.

Finally, the prevalence of disability by cause can be calculated as shown in equation (4).

4 $$ \begin{array}{l}{P}_b=\frac{N_b}{N}\\ {}{P}_d=\frac{N_d}{N}\end{array} $$

Where *P*_*b*_ is the prevalence of disabled individuals due to background, *P*_*d*_ is the prevalence of disabled individuals due to disease *d*, and *N* is the total number of individuals.

Separate models for men and women were fit and the confidence intervals for the disease prevalence, parameter estimates of the models (background disability rates and disease-specific disability rates), and the prevalence of disability by cause were estimated by the 2.5th and 97.5th empirical percentiles from 1000 bootstrap replicas sampled with replacement of equal sample size as the original data [24].

The diseases that were not significant in the additive hazards model were included in the “other” diseases group: chronic cystitis, stomach ulcer, bowel diseases, cirrhosis, gall-stones, cataract, glaucoma, migraine, thyroid problems, and skin diseases.

The statistical analysis was conducted in R, version 3.0.3 [25], using the software developed by Nusselder and Looman [4,13] to fit the additive regression model and to estimate the disability prevalence by cause. The Nelder-Mead simplex optimization technique was used [26]. Further details about the attribution method and software are described elsewhere [4,10,13].

## Results

Detailed information on the study participants are presented in Table 1. There was an oversampling of elderly individuals aged 65 years or older for both men and women in the 2004 and 2008 HIS. More than one third of elderly subjects reported low level of education (no diploma or primary school). The proportion of disabled individuals increased with age: more than half the individuals aged 80 years or older were disabled. Disability was higher among women compared to men in all age groups. It is important to notice that young individuals (15–54 years) already reported disability, although the proportion of disabled individuals in this age group was low (less than 5%) (Table 1).

Among men aged 15 to 79 years, arthritis, other diseases, back pain, and chronic respiratory diseases were the most prevalent diseases. In the oldest old men (≥80 years), arthritis, other diseases, heart attack and chronic respiratory diseases were the most prevalent diseases (Table 2). A low prevalence was observed for neurological diseases, stroke, and osteoporosis. For all diseases, the prevalence tended to increase with age, except for depression (Table 2).

**Table 2:** **Disease prevalence and disease-specific disability rates (disabling impacts), Belgium, 1997, 2001, 2004, and 2008**

**Diseases**	**Disease prevalence**								**Disabling Impact**							
	**15-54 years**		**55-64 years**		**65-79 years**		**≥80 years**		**15-54 years**		**55-64 years**		**65-79 years**		**≥80 years**	
	**%**	**95% CI**	**%**	**95% CI**	**%**	**95% CI**	**%**	**95% CI**	**Rate**	**95% CI**	**Rate**	**95% CI**	**Rate**	**95% CI**	**Rate**	**95% CI**
*Men*																
Chronic respiratory diseases	5.2	4.7; 5.7	9.7	8.1; 11.4	16.5	14.4; 18.5	20.9	17.0; 24.9	0.06	0.03; 0.08	0.12	0.07; 0.19	0.14	0.08; 0.22	0.25	0.09; 0.40
Diabetes	1.2	1.0; 1.4	6.8	5.4; 8.2	10.7	9.2; 12.3	10.4	7.5; 13.6	0.03	0.01; 0.06	0.06	0.01; 0.12	0.07	0.02; 0.14	0.12	0.03; 0.22
Cancer	0.3	0.2; 0.5	1.9	1.3; 2.6	4.7	3.5; 6.0	4.7	3.0; 6.5	0.07	0.02; 0.14	0.15	0.04; 0.30	0.18	0.05; 0.34	0.31	0.07; 0.58
Depression	4.3	3.8; 4.8	4.8	3.8; 5.8	5.3	3.7; 7.3	4.5	2.7; 6.6	0.07	0.03; 0.10	0.14	0.07; 0.24	0.17	0.08; 0.30	0.29	0.10; 0.50
Neurological diseases	0.7	0.5; 0.9	0.7	0.3; 1.1	2.0	1.1; 3.2	3.6	2.0; 5.6	0.30	0.17; 0.46	0.62	0.37; 1.03	0.74	0.43; 1.19	1.31	0.46; 2.00
Cardiovascular diseases																
Heart attack	1.1	0.9; 1.4	8.6	6.9; 10.3	14.7	12.9; 16.7	21.6	17.6; 25.4	0.09	0.05; 0.13	0.18	0.11; 0.28	0.21	0.14; 0.31	0.38	0.15; 0.54
Stroke	0.2	0.1; 0.3	1.0	0.6; 1.5	2.0	1.4; 2.8	5.6	3.2; 8.3	0.14	0.06; 0.26	0.31	0.14; 0.58	0.36	0.16; 0.66	0.64	0.22; 1.02
Musculoskeletal diseases																
Back pain	11.1	10.4; 11.9	16.9	14.8; 19.2	18.0	15.6; 20.6	13.3	10.6; 16.3	0.06	0.04; 0.08	0.13	0.08; 0.19	0.15	0.10; 0.22	0.27	0.10; 0.37
Osteoporosis	0.4	0.3; 06	1.7	1.1; 2.4	3.7	2.7; 4.8	5.7	3.7; 8.2	0.16	0.08; 0.29	0.34	0.19; 0.59	0.41	0.22; 0.71	0.72	0.30; 1.09
Arthritis	6.9	6.2; 7.5	22.8	20.4; 25.2	29.2	26.5; 31.8	34.5	30.1; 38.8	0.04	0.02; 0.06	0.09	0.05; 0.12	0.10	0.06; 0.15	0.18	0.06; 0.27
“Other” diseases	13.8	13.0; 14.7	19.1	16.9; 21.3	25.6	23.1; 28.0	33.5	29.3; 38.1	0.02	0.01; 0.03	0.04	0.01; 0.07	0.05	0.01; 0.09	0.08	0.02; 0.15
*Women*																
Chronic respiratory diseases	6.6	6.0; 7.3	8.3	7.0; 9.5	12.6	10.9; 14.3	11.3	8.8; 14.1	0.06	0.03; 0.08	0.09	0.05; 0.15	0.14	0.08; 0.21	0.26	0.14; 0.41
Diabetes	1.4	1.1; 1.7	6.0	4.8; 7.3	9.9	8.5; 11.3	8.6	7.0; 10.5	0.07	0.03; 0.12	0.12	0.06; 0.19	0.17	0.08; 0.27	0.32	0.14; 0.53
Cancer	0.8	0.6; 1.0	2.7	1.9; 3.6	4.5	3.2; 5.9	3.7	1.9; 6.3	0.08	0.02; 0.15	0.13	0.04; 0.24	0.19	0.06; 0.34	0.36	0.10; 0.69
Depression	6.7	6.1; 7.3	9.6	8.1; 11.2	9.5	8.1; 11.0	7.5	5.8; 9.7	0.06	0.03; 0.09	0.10	0.05; 0.16	0.14	0.07; 0.23	0.27	0.12; 0.45
Neurological diseases	0.7	0.5; 1.0	1.5	0.9; 2.2	1.7	1.2; 2.3	3.6	2.2; 5.2	0.22	0.11; 0.38	0.38	0.18; 0.63	0.55	0.26; 0.89	1.02	0.49; 1.73
Cardiovascular diseases																
Heart attack	0.9	0.6; 1.1	3.7	2.7; 4.9	9.7	8.2; 11.4	13.9	10.9; 17.5	0.08	0.04; 0.13	0.13	0.06; 0.21	0.18	0.09; 0.31	0.35	0.16; 0.58
Stroke	0.2	0.1; 0.4	1.2	0.6; 1.9	2.3	1.6; 3.2	5.0	3.3; 7.2	0.28	0.14; 0.48	0.48	0.25; 0.80	0.69	0.36; 1.13	1.29	0.65; 2.15
Musculoskeletal diseases																
Back pain	10.9	10.1; 11.7	20.4	18.2; 22.7	25.2	22.8; 27.5	21.4	18.4; 24.4	0.07	0.05; 0.09	0.12	0.07; 0.17	0.17	0.11; 0.23	0.32	0.19; 0.47
Osteoporosis	1.6	1.3; 1.9	11.2	9.6; 12.9	20.1	18.2; 22.2	22.1	19.1; 25.4	0.03	0.01; 0.06	0.05	0.01; 0.10	0.07	0.02; 0.14	0.14	0.03; 0.28
Arthritis	8.8	8.1; 9.5	32.8	30.3; 36.3	50.2	47.7; 52.6	55.0	51.0; 59.0	0.09	0.06; 0.12	0.15	0.11; 0.19	0.22	0.11; 0.20	0.41	0.27; 0.55
“Other” diseases	26.8	25.7; 27.9	33.5	30.9; 36.3	41.1	38.5; 43.7	44.2	40.4; 47.8	0.01	0.00; 0.02	0.02	0.00; 0.04	0.03	0.00; 0.04	0.05	0.01; 0.10

“Other” diseases included: chronic cystitis, stomach ulcer, bowel diseases, cirrhosis, gall-stones, cataract, glaucoma, migraine, thyroid problems, and skin diseases.

Among women, arthritis, other diseases, back pain and depression were the most prevalent diseases in young women (15–64 years), while the three musculoskeletal diseases (arthritis, back pain, and osteoporosis) and other diseases were by far the most prevalent diseases in elderly women (≥65 years). Neurological diseases, stroke, and cancer were the least prevalent diseases among women (Table 2).

Higher prevalence of chronic respiratory diseases, cancer, depression, osteoporosis, arthritis, and other diseases was observed for women compared to men aged 15 to 54 years. For individuals aged 55–64 years, the prevalence of previous heart attack in men was more than twice as large as the prevalence in women. However, depression, arthritis, osteoporosis, and other diseases were more prevalent among women. In the older individuals (≥65 years), chronic respiratory diseases and heart attack were more prevalent in men. Nonetheless, musculoskeletal and other diseases were more prevalent in elderly women compared to elderly men (Table 2).

**Figure 1:**
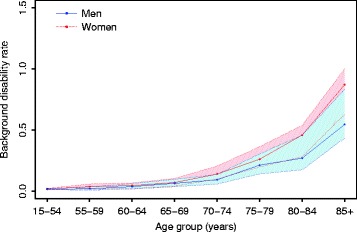
**Background disability rate by gender and age group.** Health Interview Survey, Belgium, 1997, 2001, 2004, and 2008.

Low background disability rates were observed in young individuals (<65 years), with an increasing trend over age. Although not significant, a small gender gap was observed, where higher point estimates for the background disability rates were observed among women (Figure 1).

The highest disabling impacts were observed for neurological diseases and stroke for men and women. Osteoporosis was also important for men while arthritis was among the most disabling diseases for women. Low disabling impacts were observed for other diseases, diabetes, and arthritis among men, and for other diseases, osteoporosis, and chronic respiratory diseases among women. Overall, an increase in disabling impacts with increasing age was observed. Men showed higher disabling impacts than women across all age groups for osteoporosis (Table 2).

The disability prevalence was low among young individuals (<5%) and it increased over age. A higher prevalence of disability was observed in women compared to men for all age groups, except for individuals aged 55 to 64 years (Table 3).

**Table 3:** **Absolute contribution of diseases to the prevalence of disability (per 100), Belgium, 1997, 2001, 2004, and 2008**

**Diseases**	**Age group (years)**							
	**15-54**		**55-64**		**65-79**		**≥80**	
	**Prevalence**	**95% CI**	**Prevalence**	**95% CI**	**Prevalence**	**95% CI**	**Prevalence**	**95% CI**
*Men*								
Background	1.55	1.55; 1.56	2.60	2.55; 2.66	9.67	9.37; 9.98	25.74	24.02; 27.59
Chronic respiratory diseases	0.28	0.16; 0.40	1.03	0.54; 1.59	1.92	1.02; 2.86	2.96	1.26; 5.02
Diabetes	0.03	0.01; 0.06	0.37	0.10; 0.70	0.64	0.16; 1.21	0.74	0.19; 1.57
Cancer	0.02	0.00; 0.05	0.25	0.06; 0.49	0.66	0.18; 1.21	0.79	0.21; 1.61
Depression	0.27	0.13; 0.42	0.58	0.27; 0.93	0.67	0.32; 1.16	0.67	0.27; 1.22
Neurological diseases	0.18	0.10; 0.29	0.30	0.13; 0.52	0.97	0.44; 1.73	1.80	0.72; 3.22
Cardiovascular diseases								
Heart attack	0.09	0.05; 0.15	1.32	0.76; 2.00	2.51	1.58; 3.53	4.37	2.26; 6.82
Stroke	0.03	0.01; 0.05	0.25	0.08; 0.50	0.54	0.25; 0.84	1.82	0.67; 3.44
Musculoskeletal diseases								
Back pain	0.63	0.44; 0.83	1.91	1.17; 2.72	2.18	1.51; 2.91	1.89	0.95; 2.98
Osteoporosis	0.07	0.03; 0.12	0.47	0.26; 0.71	1.09	0.54; 1.74	1.93	0.95; 3.22
Arthritis	0.26	0.15; 0.38	1.73	1.11; 2.48	2.42	1.39; 3.51	3.50	1.58; 5.86
“Other” diseases	0.24	0.08; 0.42	0.65	0.23; 1.17	0.95	0.30; 1.71	1.50	0.44; 3.02
Total disability prevalence	3.65	3.26; 4.02	11.45	9.51; 13.43	24.21	21.59; 26.71	47.71	40.23; 54.38
*Women*								
Background	1.81	1.81; 1.82	3.80	3.74; 3.85	12.02	11.64; 12.38	36.80	34.75; 39.01
Chronic respiratory diseases	0.34	0.21; 0.50	0.66	0.37; 1.01	1.26	0.71; 1.96	1.43	0.78; 2.29
Diabetes	0.09	0.04; 0.16	0.59	0.29; 0.98	1.23	0.62; 1.98	1.40	0.66; 2.25
Cancer	0.06	0.02; 0.11	0.30	0.08; 0.58	0.63	0.21; 1.11	0.68	0.16; 1.56
Depression	0.35	0.19; 0.54	0.78	0.37; 1.29	0.97	0.48; 1.56	0.99	0.45; 1.69
Neurological diseases	0.15	0.06; 0.27	0.39	0.19; 0.66	0.55	0.27; 0.88	1.32	0.65; 2.17
Cardiovascular diseases								
Heart attack	0.06	0.03; 0.10	0.38	0.17; 0.64	1.27	0.68; 2.05	2.33	1.07; 3.75
Stroke	0.06	0.02; 0.13	0.40	0.20; 0.66	0.89	0.48; 1.44	2.29	1.27; 3.54
Musculoskeletal diseases								
Back pain	0.70	0.48; 0.93	2.00	1.26; 2.93	3.13	2.07; 4.27	3.49	2.22; 4.83
Osteoporosis	0.05	0.01; 0.09	0.50	0.13; 0.92	1.15	0.30; 2.13	1.61	0.41; 2.99
Arthritis	0.72	0.50; 0.99	4.20	3.15; 5.52	8.29	6.41; 10.26	12.02	8.63; 15.46
“Other” diseases	0.30	0.07; 0.53	0.58	0.13; 1.07	0.91	0.20; 1.71	1.32	0.28; 2.53
Total disability prevalence	4.68	4.18; 5.22	14.58	12.76; 16.79	32.30	29.57; 34.88	65.68	61.47; 69.41

The disease contribution do not sum to the total disability prevalence due to rounding.

“Other” diseases included: chronic cystitis, stomach ulcer, bowel diseases, cirrhosis, gall-stones, cataract, glaucoma, migraine, thyroid problems, and skin diseases.

**Figure 2:**
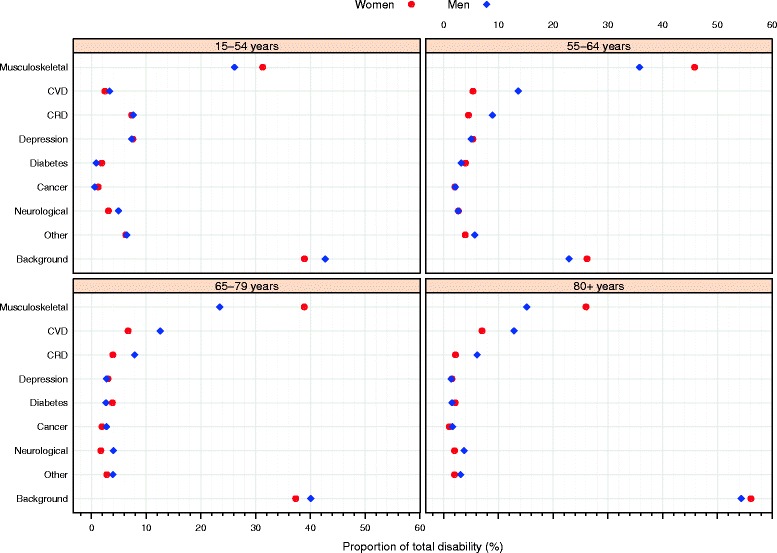
**Relative contribution of diseases to the prevalence of disability.** Health Interview Survey, Belgium, 1997, 2001, 2004, and 2008. Legend: Contributions expressed as the proportion of total disability (the sum of the cause-specific contribution across age group and gender sum to 100%). CVD – cardiovascular diseases: stroke and heart attack; CRD – chronic respiratory diseases; Musculoskeletal diseases: back pain, osteoporosis, and arthritis (rheumatoid arthritis and osteoarthritis); Neurological diseases: epilepsy and Parkinson’s disease; other diseases: chronic cystitis, stomach ulcer, bowel diseases, cirrhosis, gall-stones, cataract, glaucoma, migraine, thyroid problems, and skin diseases.

The attribution of disability to diseases depends on the disease prevalence and the disability rate of each disease. The far highest contributors to the burden of disability were musculoskeletal diseases, followed by cardiovascular diseases for men and women (Table 3, Figure 2). For all ages, musculoskeletal diseases accounted for 15 to 36% of the prevalence of disability in men and 31 to 56% in women. Cardiovascular diseases represented 13 to 14% of the disability burden in men aged 55 years or older and 7% in women aged 55 years or older. While depression was also an important cause of disability in young individuals (15–54 years), representing 7% of the disability burden, chronic respiratory diseases accounted for 7% of the disability prevalence in the youngest and 2 to 6% in the oldest-old subjects (Figure 2).

Among elderly individuals (≥65 years), the three main contributors to the disability burden – musculoskeletal, cardiovascular, and chronic respiratory diseases – represented 34 to 44% and 49 to 65% of the disability prevalence in men and women, respectively (Figure 2). It is important to notice that the contribution of musculoskeletal diseases was much larger than cardiovascular diseases for both men and women (Figure 2).

In men, back pain was the most important contributor of the musculoskeletal group among young individuals (<65 years) while arthritis was the most important contributor among the oldest men (≥65 years). In women, arthritis was the main contributor of the musculoskeletal group in all age groups. For cardiovascular diseases, heart attack was the main cause of disability in both men and women (Table 3).

Cancer and neurological diseases showed a low contribution to the prevalence of disability, accounting for less than 5% of the total disability. Furthermore, depression also had a low contribution to the prevalence of disability among elderly women and men (<3%) (Figure 2).

A large relative contribution of background was observed, especially among the youngest men (43%) and women (39%) and among the oldest old, in which more than 50% of the total disability prevalence was attributed to background (Figure 2).

A model without the interaction between diseases and disability, which provides the estimates for the total population of 15 years and older, was also fitted and included as an additional file (Additional file 2). By ignoring the interaction of age and diseases, back pain, heart attack, and arthritis were the main contributors to the disability burden in men while arthritis, back pain, and chronic respiratory diseases were the most important contributors among women (Additional file 2).

## Discussion

The major contributors to the disability burden in Belgium were musculoskeletal diseases, followed by cardiovascular and chronic respiratory diseases.

Among elderly (≥65 years) individuals, musculoskeletal diseases were the main cause of disability due to their high prevalence and moderate disabling impact, while cardiovascular diseases were also an important cause of disability because of their moderate prevalence and high disabling impact. Furthermore, chronic respiratory diseases were among the main causes of disability due to the combination of their moderate prevalence and disabling impact, showing that the impact of diseases on the disability burden depends on both the prevalence and disabling impact of the diseases [4,11]. Nonetheless, neurological diseases, which are known strong disabling diseases [14,27], presented a high disabling impact but a low prevalence, resulting in a low contribution of the disability burden in Belgium. This low contribution can also be related to the fact that dementia and Alzheimer disease, important contributors to the disability burden in the elderly, were not included in this disease group due to lack of availability in the four HIS.

The occurrence of disability in the youngest individuals (15–54 years) indicates that attention should also be given to this group, although young adults are often perceived as “healthy”. More important, depression was identified as one of the main causes of disability in young individuals. Other studies already showed that depression is one of the most disabling diseases related to loss of quality of life among the mental disorders [27], which can also affect the performance of ADLs [28,29]. Unipolar depressive disorders were the main cause of years lost due to disability in the population of 15 to 24 years of age worldwide [30]. Our findings highlight the importance of including this disease in the surveys and in the analysis of cause-specific disability.

A large contribution of background to the disability burden may reflect that: (i) disability can occur without any disease, (ii) diseases in the survey can be underreported, (iii) diseases and conditions may no longer be present in the year preceding the interview (e.g., consequences of accidents), (iv) the causes of disability (diseases or conditions) were not included in the analysis [11], or (v) diseases may be underdiagnosed. The relative background contribution was larger in the youngest and oldest age groups, which might indicate that the main causes of disability in these groups were not included in the list of chronic diseases explored during the surveys, e.g. permanent consequences of accidents and injuries in the youngest and dementia and Alzheimer disease in the oldest individuals [10,13]. Aging-related functional loss may also explain the large background contribution observed among elderly [4,10].

The comparability of our results with previous studies that also used the attribution method [3,10,11,13-15] is limited mainly due to differences in the disability definition, the diseases included in the analysis, the target population, and the survey methodology.

In the present study, disability was defined based on six ADL functions and mobility limitations, including moderate and severe disability levels. The use of this definition is supported by the fact that ADLs are considered basic tasks to survival. Hence, difficulties in performing these activities suggests serious health problems [31].

The first study that used the attribution method, analysed the data of the 1997 Belgian HIS. In this study, disability was defined based on four moderate or severe functional limitations from the short-form 36 and included only individuals age 30 years and older [13]. Other three studies used Dutch data and included only severely disabled individuals. While in the first study [10] data from 1990 to 1994 surveys were analysed and the disability indicator was based on ADL, communication and sensorial limitations, the two most recent studies used pooled data from 2001 to 2007. In these two studies, two different disability definitions were used: (i) based on ADL and mobility limitations [11], which is the most comparable with the present study, and (ii) based on hearing, vision, and mobility limitations [3]. The study conducted in Germany [14] defined disability based on the health assessment questionnaire disability index, which also includes ADL limitations, and was restricted to individuals aged 65 years or older. In contrast to previous studies, the study conducted in China [15] focused on the causes of impairments, such as intellectual deficits, hearing and vision limitations and not on the causes of ADL limitations. Thus, we did not include the results of the Chinese study in the comparison.

We did not compare our results with the Global Burden of Diseases study (GBD 2010) [32], also due to methodological differences. In the GBD 2010, the disability burden was assessed by the population health metric called years lived with disability (YLD), which is a function of the prevalence of disease sequelae and disability weights [32]. The disability weights were used to quantify the severity of disease sequelae and were based on survey responses of the general population [32]. In contrast, the attribution method used in this study estimates the disability prevalence by cause as a function of the disease prevalence and the disease-specific disability rates. The disease-specific disability rates are estimated from the additive regression model. Another difference between the two methods is the outcome of interest: while the GBD 2010 focused on years lived with disability, which can be considered a measure of healthiness of years lived with a disease [32], the attribution method estimates the disability burden based on the disability prevalence by cause, where disability was defined based on functional and mobility limitations.

Despite the methodological differences, musculoskeletal and cardiovascular diseases were among the main causes of disability in all previous studies [3,10,11,13-15,32,33].

There are several limitations in the present study that should be considered when interpreting the results. Temporal bias might have occurred as a consequence of the use of cross-sectional data and disability may be incorrectly attributed to disease when disability onset preceded disease onset [11]. Although selection bias can occur due to high non-response rates, it may be reduced with the use of post-stratification weights under the assumption that non-responders are not different from responders with similar demographic characteristics [11,34]. Also, lack of information of possible important causes of disability such as dementia and injuries, the use of self-reported diseases, and underdiagnoses of disease may have resulted in an overestimation of the background contribution. The validity of self-reported diseases is specific for each disease [35]: while a good validity was previously reported for diabetes, hypertension, stroke [36], cancer [37], and asthma [38], a poor validity was observed for self-reported thyroid problems [39] and arthritis [40]. Additionally, previous studies showed a moderate to weak relationship between self-reported and performed-based ADL [41] and mobility limitations [42] in the elderly. One possible explanation for these differences is that performance-based measures often do not reflect adaptations made to living situations of a person [41]. Moreover, our disability definition was restricted to functional and mobility limitations. Although other measures of disability, such as instrumental activities of daily living, cognitive impairments, and sensory limitations can also be important in the disablement process [43], we did not include them because they were not consistently available in the four Belgian HIS.

The use of a large age interval for the young individuals (15–54 years) assumes that the disease-specific rates and respective contributions were constant in this age group. Although the disease-specific rates tended to increase with age, a model with smaller age intervals (5 years) was initially fitted (data not shown). However, the disabling impacts were very small and similar for individuals younger than 55 years. Therefore, this larger age group was used in order to have a more parsimonious model.

Recently, a modification of the average attribution fraction has been proposed to account for the coexistence of diseases in the mortality analysis [44,45]. The attribution method also allows the attribution of disability to more than one disease by including interaction terms between diseases as the *X*_*di*_ variable in the model [4]. However, no interaction terms between diseases were included in our analysis due to the large number of diseases and the large sample size, which would result in a computationally intensive task. By ignoring the interaction of diseases, it is possible that both under and overestimation of the attribution of disability to diseases occurred, similar to what has been shown in the mortality analysis [44,45].

Furthermore, the exclusion of individuals for whom disease or disability were missing suggests that the prevalence of disability might be underestimated, as most of these individuals were women, elderly, and low educated, who are known to have high disability prevalence [13,46]. However, since this proportion was small (8%), the impact on the disability prevalence may not be important. In addition, one could argue that the use of pooled data from the four HIS may have biased the results due to changes in the disability prevalence over the 1997 to 2008 period. Nevertheless, as shown in Additional file 3, the disability prevalence seemed to be stable across gender and age groups over the period. The use of pooled data allowed increasing the power of the statistical analysis, especially in the older age groups.

An important strength of this study is that the attribution method applied here takes into account that subjects without reported diseases can be disabled (“background”). Additionally, the additive property of rates (background and disease-specific disability rates) allows partitioning the disability prevalence into additive disease contributions in the presence of comorbidity [10].

Added values of this study include the analysis of depression, which showed to be one of the major causes of disability in young individuals and the inclusion of institutionalized population in the survey sample, allowing a better generalization of the results to the Belgian elderly population.

## Conclusions

In summary, this study identified musculoskeletal, cardiovascular, and respiratory diseases as the main causes of disability in Belgium across all age groups in both men and women. Besides these conditions, depression was also an important cause of disability, especially among young individuals. Intervention policies should focus on the prevention of disease onset and reduction of disabling impacts of these diseases.

It would be interesting to investigate whether the model can be extended to incorporate a multinomial response (disability level), allowing the comparison of disability prevalence by cause according to severity level (e.g., moderate and severe).

Further research should explore the role of risk factors for chronic diseases, such as smoking and obesity, in the cause-specific disability prevalence.

## Additional files

Additional file 1:
**Comparison of the characteristics of individuals with missing data on diseases or disability and individuals with complete data in the analysis.** Health Interview Survey, Belgium, 1997, 2001, 2004, and 2008. Additional file 2:
**Disease prevalence, disease-specific disability rate, and absolute contribution of diseases to the prevalence of disability (per 100) in the population aged 15 years and older.** Health Interview Survey, Belgium, 1997, 2001, 2004, and 2008. Additional file 3:
**Prevalence of disability by gender, survey year, and age group.** Health Interview Survey, Belgium, 1997, 2001, 2004, and 2008.

## Abbreviations

ADL: Activity of daily living
CRD: Chronic respiratory diseases
CVD: Cardiovascular diseases
GBD: Global burden of diseases
HIS: Health interview survey
OR: Odds ratio
YLD: Years lived with disability
