# Supplementary material for: Contribution of chronic diseases to the disability burden in a population 15 years and older, Belgium, 1997–2008
Source: BMC Public Health. 2015 Mar 7;15:229. doi: 10.1186/s12889-015-1574-z (PMC4361141; doi:10.1186/s12889-015-1574-z)
Supplement: Additional file 1: — Comparison of the characteristics of individuals with missing data on diseases or disability and individuals with complete data in the analysis. Health Interview Survey, Belgium, 1997, 2001, 2004, and 2008. [file 12889_2015_1574_MOESM1_ESM.docx]

Additional file 1 Comparison of the characteristics of individuals with missing data on diseases or disability and individuals with complete data in the analysis. Health Interview Survey, Belgium, 1997, 2001, 2004, and 2008.

| Characteristics | Individuals with missing data | | Individuals with complete data | | p-value |
| --- | --- | --- | --- | --- | --- |
|  | N | % | N | % |  |
| Gender |  |  |  |  |  |
| Men | 1614 | 43.0 | 17034 | 47.5 | <0.001 |
| Women | 2136 | 57.0 | 18803 | 52.5 |  |
| Age group (years) |  |  |  |  |  |
| 15-54 | 1581 | 42.2 | 22505 | 62.8 | <0.001 |
| 55-64 | 729 | 19.4 | 4499 | 12.6 |  |
| 65-79 | 918 | 24.5 | 5641 | 15.7 |  |
| ≥80 | 522 | 13.9 | 3192 | 8.9 |  |
| Level of education |  |  |  |  |  |
| No diploma | 72 | 2.1 | 514 | 1.6 | <0.001 |
| Primary | 754 | 22.3 | 5609 | 17.6 |  |
| Secondary | 1476 | 43.6 | 14157 | 44.4 |  |
| Tertiary | 1085 | 32.0 | 11619 | 36.4 |  |

The p-value was obtained the by χ^2^ test.
