# Supplementary material for: Contribution of chronic diseases to the disability burden in a population 15 years and older, Belgium, 1997–2008
Source: BMC Public Health. 2015 Mar 7;15:229. doi: 10.1186/s12889-015-1574-z (PMC4361141; doi:10.1186/s12889-015-1574-z)
Supplement: Additional file 2: — Disease prevalence, disease-specific disability rate, and absolute contribution of diseases to the prevalence of disability (per 100) in the population aged 15 years and older. Health Interview Survey, Belgium, 1997, 2001, 2004, and 2008. [file 12889_2015_1574_MOESM2_ESM.doc]

Additional file 2: Disease prevalence, disease-specific disability rate, and absolute contribution of disease to the prevalence of disability (per 100) in the population aged 15 years and older. Health Interview Survey, Belgium, 1997, 2001, 2004, and 2008.

| Diseases | Disease prevalence | | Disabling impact | | Absolute contribution to the disability prevalence | |
| --- | --- | --- | --- | --- | --- | --- |
|  | % | 95%CI | Rate | 95%CI | Prevalence | 95%CI |
| *Men* |  |  |  |  |  |  |
| Background | - | - | - | - | 4.23 | 3.73; 4.72 |
| Chronic respiratory diseases | 7.6 | 7.1; 8.1 | 0.06 | 0.03; 0.10 | 0.39 | 0.18; 0.64 |
| Diabetes | 3.3 | 3.0; 3.6 | 0.05 | 0.01; 0.10 | 0.14 | 0.03; 0.28 |
| Cancer | 1.2 | 1.0; 1.4 | 0.16 | 0.03; 0.30 | 0.14 | 0.03; 0.28 |
| Depression | 4.5 | 4.0; 4.9 | 0.08 | 0.04; 0.12 | 0.31 | 0.15; 0.47 |
| Neurological diseases | 1.0 | 0.8; 1.2 | 0.43 | 0.24; 0.66 | 0.30 | 0.16; 0.47 |
| Cardiovascular diseases |  |  |  |  |  |  |
| Heart attack | 4.3 | 4.0; 4.7 | 0.20 | 0.12; 0.28 | 0.68 | 0.42; 0.94 |
| Stroke | 0.7 | 0.6; 0.8 | 0.33 | 0.15; 0.53 | 0.16 | 0.08; 0.26 |
| Musculoskeletal diseases |  |  |  |  |  |  |
| Back pain | 12.7 | 12.0; 13.5 | 0.08 | 0.05; 0.10 | 0.88 | 0.63; 1.16 |
| Osteoporosis | 1.2 | 1.0; 1.4 | 0.39 | 0.23; 0.60 | 0.33 | 0.20; 0.48 |
| Arthritis | 12.3 | 11.7; 13.1 | 0.06 | 0.03; 0.09 | 0.65 | 0.37; 0.94 |
| “Other” diseases | 16.5 | 15.7; 17.2 | 0.02 | 0.00; 0.03 | 0.26 | 0.04; 0.51 |
| Total disability prevalence | - | - | - | - | 8.47 | 7.91; 8.98 |
| *Women* |  |  |  |  |  |  |
| Background | - | - | - | - | 7.07 | 6.42; 7.74 |
| Chronic respiratory diseases | 8.0 | 7.4; 8.5 | 0.05 | 0.03; 0.09 | 0.37 | 0.19; 0.58 |
| Diabetes | 3.6 | 3.3; 3.9 | 0.11 | 0.05; 0.19 | 0.32 | 0.14; 0.53 |
| Cancer | 1.7 | 1.5; 2.1 | 0.13 | 0.04; 0.24 | 0.19 | 0.06; 0.33 |
| Depression | 7.5 | 7.0; 8.0 | 0.05 | 0.02; 0.09 | 0.35 | 0.15; 0.56 |
| Neurological diseases | 1.1 | 0.9; 1.3 | 0.33 | 0.14; 0.58 | 0.27 | 0.12; 0.46 |
| Cardiovascular diseases |  |  |  |  |  |  |
| Heart attack | 3.2 | 2.8; 3.6 | 0.14 | 0.07; 0.25 | 0.35 | 0.16; 0.55 |
| Stroke | 0.9 | 0.7; 1.1 | 0.63 | 0.37; 0.96 | 0.35 | 0.21; 051 |
| Musculoskeletal diseases |  |  |  |  |  |  |
| Back pain | 14.7 | 14.0; 15.4 | 0.08 | 0.05; 0.11 | 1.05 | 0.67; 1.41 |
| Osteoporosis | 6.6 | 6.2; 7.1 | 0.06 | 0.02; 0.12 | 0.34 | 0.10; 0.61 |
| Arthritis | 20.3 | 19.5; 21.1 | 0.15 | 0.11; 0.18 | 2.37 | 1.77; 2.97 |
| “Other” diseases | 30.6 | 29.7; 31.6 | 0.01 | 0.00; 0.02 | 0.31 | 0.04; 0.62 |
| Total disability prevalence | - | - | - | - | 13.33 | 12.67; 14.00 |

The disease contribution do not sum to the total disability prevalence due to rounding.

“Other” diseases included: chronic cystitis, stomach ulcer, bowel diseases, cirrhosis, gall-stones, cataract, glaucoma, migraine, thyroid problems, and skin diseases. Background disability rates not presented because they were modelled per age group (15-54, 55-59, 60-64, 65-69, 70-74, 75-79,80-84, ≥85 years).
