# Supplementary figures and images for: Contribution of chronic diseases to the disability burden in a population 15 years and older, Belgium, 1997–2008
Source: BMC Public Health. 2015 Mar 7;15:229. doi: 10.1186/s12889-015-1574-z (PMC4361141; doi:10.1186/s12889-015-1574-z)

Men

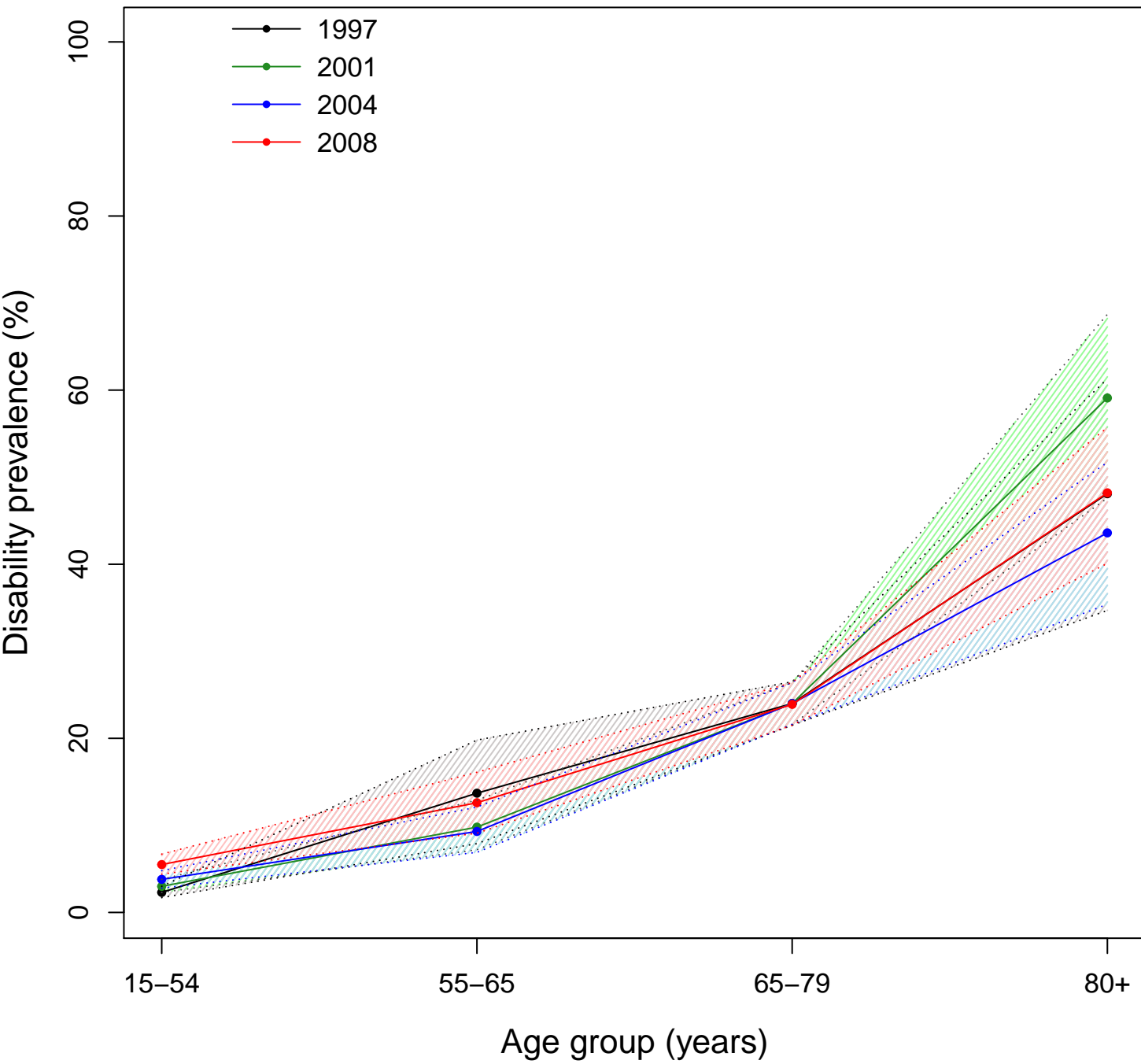

Women

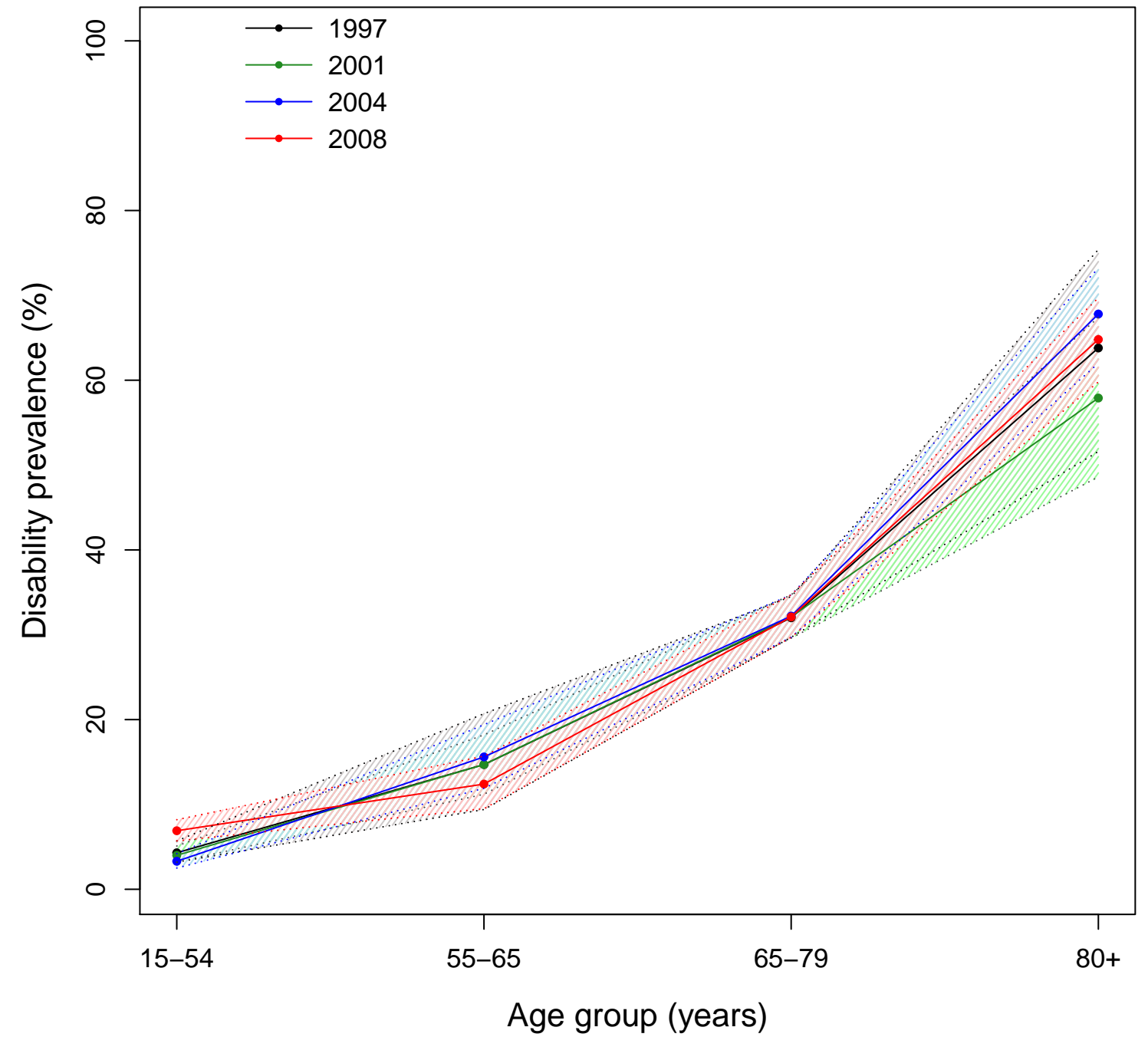

Supplement: Additional file 3: — Prevalence of disability by gender, survey year, and age group. Health Interview Survey, Belgium, 1997, 2001, 2004, and 2008. [file 12889_2015_1574_MOESM3_ESM.pdf]
